# Synthesis and Characterization of UV-Curable Resin with High Refractive Index for a Luminance-Enhancing Prism Film

**DOI:** 10.3390/polym17010076

**Published:** 2024-12-30

**Authors:** Jin Han Song, Seung-Mo Hong, Seok Kyu Park, Hyeok Ki Kwon, Seok-Ho Hwang, Jong-Min Oh, Sang-Mo Koo, Giwon Lee, Chulhwan Park

**Affiliations:** 1 Department of Chemical Engineering, Kwangwoon University, 20, Kwangwoon-ro, Nowon-gu, Seoul 01897, Republic of Korea; jhssong75@shinatnc.com (J.H.S.); david981228@gmail.com (H.K.K.); 2 R&D Center, SHIN-A T&C, 184 Gasan Digital 2-ro, Geumcheon-gu, Seoul 0851, Republic of Korea; hsm@shinatnc.com (S.-M.H.); skpark@shinatnc.com (S.K.P.); 3 School of Polymer System Engineering, Dankook University, 152 Jukjun-ro, Suji-gu, Yongin 16890, Republic of Korea; bach@dankook.ac.kr; 4 Department of Electronic Materials Engineering, Kwangwoon University, Seoul 01897, Republic of Korea; jmoh@kw.ac.kr (J.-M.O.); smkoo@kw.ac.kr (S.-M.K.)

**Keywords:** thiol–acrylate reaction, high refractive index, TFT-LCD, backlight, imprint, prism film, luminance

## Abstract

A novel monomer, 9-bis[4-(2-hydroxyethoxy)phenyl]fluorene di(mercaptopropionate), with a highly refractive index, purity, and excellent UV-curable properties, is synthesized through an optimized Fischer esterification process, reacting 9,9-bis[4-(2-hydroxyethoxy)phenyl]fluorene with 3-mercaptopropionic acid. The structural characterization of this monomer is performed using Fourier-transform infrared spectroscopy, nuclear magnetic resonance spectroscopy, high-performance liquid chromatography, and liquid chromatography-mass spectrometry. The synthesis conditions are optimized using a design-of-experiments approach. UV-curable resins are obtained by incorporating the synthesized monomer as the thiol component. The effects of thiol content on the UV-curing behavior, refractive index, shrinkage, adhesion to the polyethylene terephthalate (PET) foil, and viscoelastic recovery are examined. The thermal properties are assessed using differential scanning calorimetry and thermogravimetric analysis. Field-emission scanning electron microscopy confirms the successful replication of the prism film. In edge-lit light-emitting diode (LED) backlight units, the prism film showed increased luminance with higher thiol monomer content in the UV-curable resin while maintaining stable color coordinates. This novel highly refractive index monomer can be utilized in luminance-enhancing prism films, thereby contributing to future innovations in the display film industry.

## 1. Introduction

For decades, high-refractive index transparent materials have received significant attention in various advanced solid-state optoelectronic research fields, such as flat panel displays, imaging sensors, photonic circuits, and light-emitting diodes (LEDs) [1,2,3,4]. In particular, UV-curable resin systems for high-refractive index materials have numerous advantages over thermally cured systems, including shorter curing times, lower energy consumption, and minimal volatile organic solvents, making them suitable for broad applications [5,6,7,8,9,10].

A standard approach to increase the refractive index is to introduce substituents with high molar refraction, low molar volume, and high density, as described by the classical Lorentz–Lorenz equation [11,12,13]. Based on this theory, high-refractive index monomers for UV-curable resins can increase the refractive index by employing structures that include aromatic parts or halogen and sulfur atoms and by adding inorganic fillers. However, the aromatic portion of the monomer increases its viscosity and birefringence. Additionally, bonding halogen atoms to the monomer may lead to undesirable dark colors and potentially toxic environmental pollutants, such as strong acids generated during combustion. Achieving high optical transparency in inorganic/organic hybrid systems can be challenging because of the easy agglomeration within the polymer, attributed to their high specific surface energy and inherent hydrophilic characteristics [14,15,16]. Nonetheless, sulfur-substituted monomers can provide high transparency, high permittivity, and strong adhesion to substrates [11,17,18,19,20,21]. Recently, aromatic group-based monomers modified by sulfur atoms have been actively researched for applications aimed at increasing the refractive index of coating layers in optical films [22,23,24].

This study designed a new monomer comprising fluorene derivatives and terminal thiol groups, synthesized for use in a UV-curable resin system for prism sheet production. The new monomer, 9-bis[4-(2-hydroxyethoxy)phenyl]fluorene di(mercaptopropionate) (BPEF-DMP), was synthesized through a Fischer esterification reaction using 9,9-bis[4-(2-hydroxyethoxy)phenyl]fluorene (BPEF) and 3-mercaptopropionic acid (3-MPA), generating water as a byproduct under catalytic acidic conditions. To optimize the reaction conditions, a design of experiments approach utilizing a factorial design was employed to determine the primary factors that would allow for increased purity and to establish optimal synthesis conditions. Considering benzene rings have a much higher refractive index than saturated aliphatic rings with the same number of carbon atoms, BPEF-DMP was used to increase the refractive index of the monomer. Furthermore, the terminal thiol groups of the monomer can be introduced into a UV-curable resin system through thiol–acrylate click reactions, enhancing the refractive index of the resin system. Finally, the synthesized monomer was introduced to produce prism films with excellent luminance and physical properties. The fundamental characteristics of the resin composition were investigated based on the synthesized thiol monomer content.

## 2. Materials and Methods

### 2.1. Materials

To synthesize 9-bis[4-(2-hydroxyethoxy)phenyl]fluorene di(mercaptopropionate) (BPEF-DMP), 9,9-bis[4-(2-hydroxyethoxy)phenyl]fluorene (BPEF), 3-mercaptopropionic acid (3-MPA), and p-toluenesulfonic acid (p-TSA) were purchased from TCI (Tokyo, Japan) and used without further purification. Toluene was obtained from Samcheon Chemical (Seoul, Korea), dried over sodium sulfate, filtered, and distilled at 110 °C before use. The photoinitiator diphenyl(2,4,6–trimethylbenzoyl)phosphine oxide (TPO) was purchased from IGM Resins (Waalwijk, The Netherlands) (Omnirad TPO G) and used without purification. The polythiol compound trimethylolpropane tris(3-mercaptopropionate) (TMPMP) was provided by SHIN-A T&C (Seoul, Korea) (ST-032) and used without purification. The acrylate compound ethoxylated (7.5 EO/phenyl) 9,9-bis(4-hydroxyphenyl)fluorene diacrylate (BPEA-EDA; ethylene oxide/phenol ratio = 7.5) was obtained from Green Chemical (Seosan, Korea) (product name: KOMERATE D154), and benzyl acrylate (BZA) was purchased from Osaka Organic Chemical Industry (Osaka, Japan) (product name: Viscot #160); both were used without further purification. The polyethylene terephthalate (PET) film used in this study, with a thickness of 188 μm and primer treatment on one side, was purchased from SKC (Seoul, Korea) (V7611 grade).

### 2.2. Synthesis of BPEF-DMP

BPEF, 3-MPA, p-TSA, and toluene were introduced into a reaction vessel equipped with a mechanical stirrer, thermometer, and Dean–Stark trap with a cooling condenser. The mixture was stirred at 100 rpm and refluxed (approximately 110–115 °C) to drive the esterification reaction, and the external water byproduct was removed. The reaction was considered complete when the amount of removed water reached the theoretical value. After cooling to room temperature, 10% NaOH aqueous solution was added, and the mixture was transferred to a separatory funnel, where it was neutralized to pH 7. The unreacted 3-MPA was removed by separation into an aqueous phase. After removing the aqueous layer, the organic phase was washed thrice with distilled water and dried over anhydrous magnesium sulfate. Toluene was subsequently removed by rotary evaporation, and the product was further dried in a vacuum oven for three days to completely remove the toluene. The resulting product was obtained as a colorless viscous liquid with 95% yield.

### 2.3. Preparation of Prism Replica Mold

A commercially available prism film composed of a UV-curable resin on PET was used as the master mold. The prism film of the master mold has a pattern with grooves that are 50 µm in width and 25 µm in height. A polydimethylsiloxane (PDMS) replica mold of the prism pattern was fabricated using the Sylgard 184 silicone elastomer kit (Dow Chemical, Derbyshire, UK) via soft lithography method [25,26]. First, the master mold was attached to a flat surface, and a homogeneous mixture of Sylgard 184 and 10 phr of curing agent was evenly poured over the master mold. This mixture was subsequently placed in a vacuum chamber for 2 h to degas, and subsequently cured on a hot plate at 120 °C for 5 h. After curing, the PDMS soft mold was carefully separated to produce a soft mold for the large-scale replication of the prism film.

### 2.4. Preparation of Prism Film

The prism film was fabricated using an imprinting process. The PDMS replica mold was attached to a glass plate, and the UV-curable resin was poured into the PDMS replica mold. The mold was then covered with a PET foil, and the layers were laminated using a laminator. The UV-curable resin between the laminated films was cured using a metal halide lamp (LF-100/800-A, Lichtzen, Gunpo, Korea) to form a pattern. The irradiated light dose, measured using a radiometer, was 720 mJ/cm. After curing, the PDMS replica mold and PET foil were separated, and the cured layer that adhered to the PET formed a prism film.

### 2.5. Characterization and Instruments

#### 2.5.1. Analysis of the Chemical Structure of BPEF-DMP

To analyze the functional groups, the Fourier transform infrared (FT-IR) spectrum was measured using an FT/IR-4100 spectrophotometer (Jasco, Tokyo, Japan) in transmission mode under air conditions, with a resolution of 8 cm^−1^ and 20 scans. Liquid chromatography-mass spectrometry (LC-MS) was used to accurately identify the impurities and chemical structures of the synthesized compounds. Acetonitrile and water were used as the mobile phases, and a gradient of acetonitrile concentration from 40% to 70% was applied over 0–20 min. The compounds were separated on a Shim-pack FC-ODS column (Shimadzu, Kyoto, Japan) using an LCMS-2020 spectrometer (Shimadzu), and the mass values of each peak were analyzed to predict the structure. High-performance liquid chromatography (HPLC) was used for purity analysis. A mixture of acetonitrile and water was used as the eluent, and KH_2_PO_4_ (0.01 mol) was added as the buffer solution. An acetonitrile concentration gradient from 40% to 70% was applied over 0–18 min, and the analysis was performed using a ZORBAX C18 column (Agilent, Santa Clara, CA, USA) on an Agilent 1220 Infinity HPLC system.

To quantitatively analyze the number of mercaptan groups in the synthesized BPEF-DMPs, an end-group analysis method (SH value, g/eq.) was conducted. A 0.1 g sample of the synthesized BPEF-DMP was placed in a beaker, and 25 mL of chloroform was added, followed by stirring for 10 min. Subsequently, 10 mL of methanol was added and stirred for another 10 min. This solution was titrated with 0.1 N iodine standard solution (FUJIFILM Wako Pure Chemical, Osaka, Japan), and the endpoint was determined by the color change from yellow to colorless. The amount of 0.1 N iodine standard solution consumed was recorded, and the SH value (g/eq.) was calculated using the following equation:

(1) $$SH\;Value\left(\frac{g}{eq}\right)=\frac{Sample\;weight\left(g\right)}{C\cdot V}.$$

where, C was the concentration of iodine solution (eq./L), and V was the consumed volume of iodine solution (L)

#### 2.5.2. Characterization of Compositions

The refractive index of the composition was measured at 25 °C using an Abbe refractometer NAR-1T-1212 (ATAGO, Tokyo, Japan), with a D-line (589 nm) light source. After curing the solid specimen, a suitable amount of monobromonaphthalene solution was applied to the prism, and the measurements were conducted. The viscosity of the sample was measured using a rotational viscometer (DV-II+ Cone–Plate Viscometer, Brookfield, Middleboro, MA, USA) at 40 °C, with spindle number 51, adjusting the spindle rotation speed to achieve 50% to 70% of the torque range. The densities of the liquid and solid samples were measured at 25 °C using an electronic densimeter (CD-V3, CAS, Yangju, Korea). Adhesion tests were conducted according to the ASTM D3359 [27] guidelines. The coated area of the manufactured prism film was cross hatched with a 10 × 10 scriber, and after applying a 3 M 610 tape to the cross-hatched area, the tape was pulled off quickly to perform the adhesion test of the cured film. The cross hatch was visually assessed, and adhesion was rated from 5 B (no peeling) to 0 B (65% peeling).

To confirm the chemical changes in the composition before and after curing, the FT-IR spectra were analyzed to observe the differences in the characteristic peaks. The measurements were performed on an FT/IR-4100 spectrophotometer (Jasco) with a resolution of 8 cm^−1^ and 20 scans, using the attenuated total reflectance (ATR) method to observe changes in the functional groups before and after curing. For optical property measurements based on the curing conditions of the prism film, the transmittance was measured in the 300–700 nm wavelength range using a UV/Vis spectrophotometer (UV-2450, Shimadzu). To observe the color changes, a spectrophotometer colorimeter CM-3700A (Konica Minolta, Tokyo, Japan) was used with a white ceramic plate (CR-A43) for calibration. The total transmittance and haze were measured to assess the opacity and light transmission. The curing reaction heat was monitored using photo-differential scanning calorimetry (DSC) to measure the curing speed and reaction characteristics during UV curing. A PerkinElmer DSC 8500 (PerkinElmer, Shelton, CT, USA) and OmniCure S2000 (Excelitas Technologies, Pittsburgh, PA, USA) instrument was used for UV light irradiation. The UV light source was a mercury lamp with a power of 200 W and emission wavelength between 250 and 450 nm. The light intensity was adjusted through an aperture to a dose of 10 mW/cm^2^, and measurements were conducted under isothermal conditions at 25 °C without introducing additional gases.

To analyze the thermal properties of the cured materials, DSC was performed using a DSC 1 device (Mettler Toledo, Columbus, OH, USA) to measure the glass transition temperatures (Tg) of the samples. The scan was performed under nitrogen atmosphere at a heating rate of 10 °C/min. Thermogravimetric analysis (TGA) was conducted using a Mettler TGA/SDTA 851e system with a heating rate of 10 °C/min over a temperature range of 30–600 °C, under a nitrogen atmosphere, to measure weight loss.

#### 2.5.3. Characterization of Prism Films

Field emission scanning electron microscopy (FE-SEM) was used to confirm the shape of the prism film. An S-4700 model from Hitachi High-Tech (Tokyo, Japan) was employed, and the sample was coated with platinum. The cross-sectional and top-view shapes of the prism films were observed at various magnifications. To assess the mechanical properties of the prism film, the elastic recovery was measured using a Fischerscope H100C XYp microindenter (Sindelfingen, Germany). The elastic recovery of the cured layer with a prism shape was measured using a microindenter. A load of 24.5 mN/s was applied for 10 s to induce displacement, denoted as L1. The same load was removed at the same speed for another 10 s, and the recovery displacement, denoted as L2, was measured. The elastic recovery was calculated as the ratio of L1 to L2. In addition, a custom-built prism strength tester was used to measure the shape of the prism film. Various polarizing films with different haze values were placed on top of the prism film, and a weight was placed on the polarizing film. The maximum weight that could be applied without damaging the shape of the prism film was recorded as the strength of the film (g).

A prototype edge-lit LCD backlight unit (BLU) (100 mm × 100 mm) was first fabricated to investigate the effect of different prism film compositions on the luminance and color coordinates of the BLU. The LCD BLU comprises a light guide plate (LGP), a diffuser film assembled with an LED light source, and a diffuser film that homogenizes the light from the light source through the emission surface of the BLU. The prism film fabricated in this study was laminated on top of an LCD BLU. The luminance was measured using a luminance colorimeter BM-7A (TOPCON, Tokyo, Japan). The distance between the luminance meter and backlight was fixed at 100 cm, and the measurements were conducted in a dark room to avoid light interference.

## 3. Results and Discussion

### 3.1. Chemical Structure of BPEF-DMP

**Figure 1:**
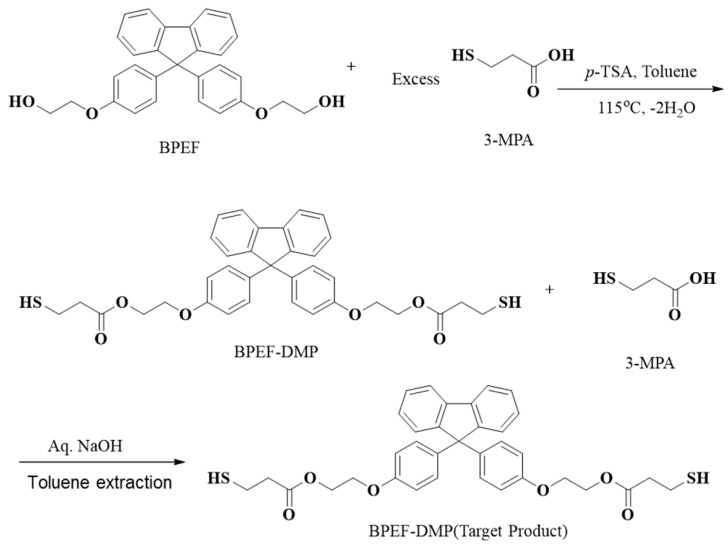
Synthesis and purification of BPEF-DMP.

**Figure 2:**
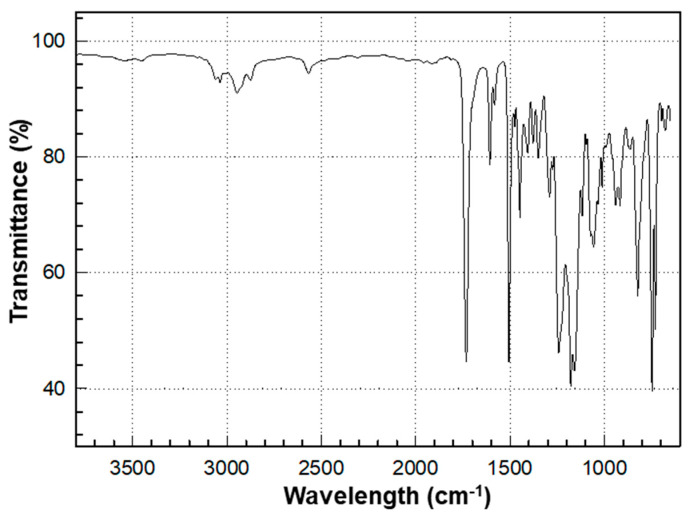
FT-IR spectrum of BPEF-DMP.

The overall chemical reaction for the synthesis of BPEF-DMP is shown in Figure 1. The BPEF-DMP monomer, designed to increase the refractive index, was synthesized through Fischer esterification using BPEF and 3-MP. Figure 2 shows the FT-IR spectrum of synthesized BPEF-DMP. As shown in Figure 2, the broad band around 3600–3200 cm^−1^, corresponding to the stretching vibration of the hydroxyl (-OH) group in BPEF, disappeared. Meanwhile, the stretching vibration peaks at 1180 and 1130 cm^−1^ were attributed to the ether group (-C-O-) in BPEF-DMP, and the peak at 1719 cm^−1^ confirmed the formation of an ester group (-O-C=O) generated by the reaction of BPEF and 3-MPA. Additionally, a characteristic stretching vibration of the -SH group was observed at 2559 cm^−1^, confirming the presence of thiol groups in BPEF-DMP.

**Figure 3:**
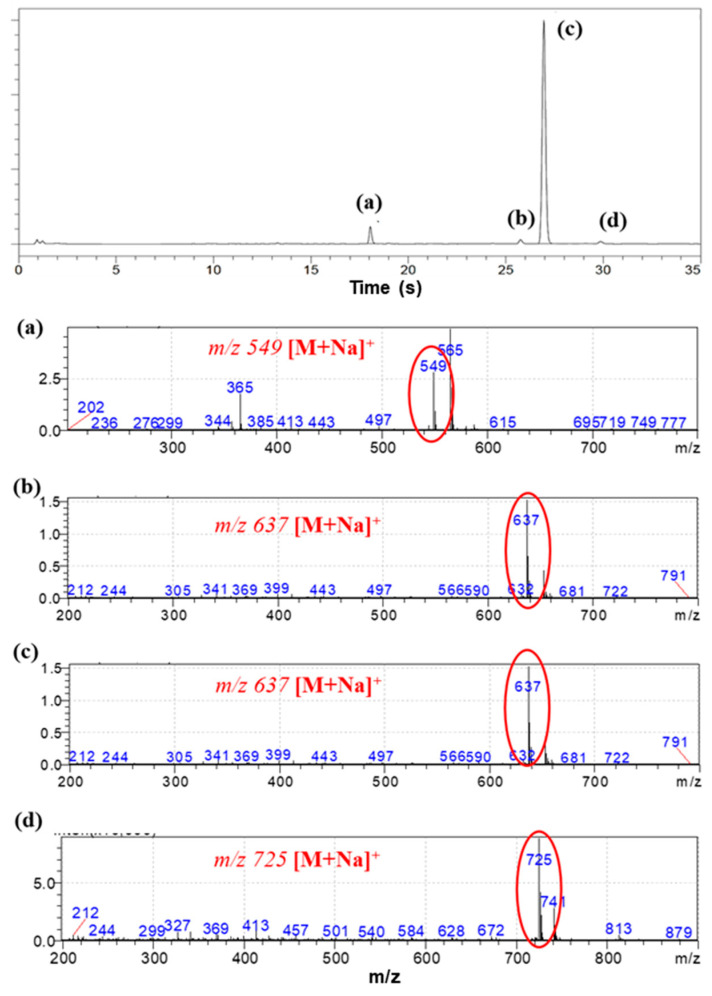
(**a**–**d**) LC-MS chromatogram and mass spectra for the eluted peak of BPEF-DMP.

**Figure 4:**
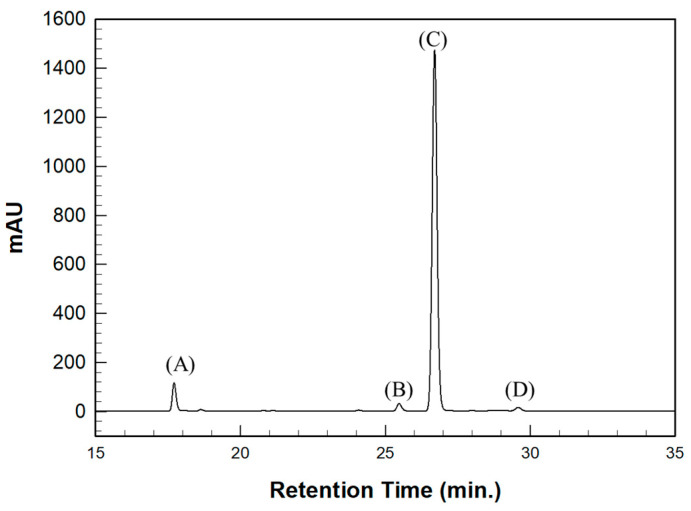
HPLC chromatography of synthesized BPEF-DMP. (A) Unreacted product, (B) Unreacted product and over-reaction, (C) Target product, and (D) Over-reaction.

The SH value of the synthesized BPEF-DMP was found to be 340.77 g/eq., and the refractive index at 25 °C was a relatively high 1.6292. LC-MS analysis was performed to detect impurities in the synthesized BPEF-DMP, including byproducts. Potential impurities were identified by comparing the retention times (RT). Using the ESI analysis of the LC-MS data, the mass values were used to predict the chemical structures, as shown in Figure 3 and Table 1. Accordingly, the expected chemical structures of the peaks observed in the HPLC analysis of BPEF-DMP matched the LC-MS data, as shown in Figure 4.

As indicated in Figure 3 and Figure 4, the peak at RT 27 min in the positive ion mode (*m*/*z* 637 [M+Na]^+^) matched the mass value of the sodium adduct of the desired BPEF-DMP. The impurities that could be identified by LC-MS were divided into two categories of by-products: unreacted byproducts, which contained unreacted hydroxyl groups from BPEF-DMP, and overreaction byproducts, which contained thiol–ester linkages. The byproducts are listed in Table 1.

### 3.2. Compositions for Prism Film

#### 3.2.1. Preparation and Characterization of the Composition

**Figure 5:**
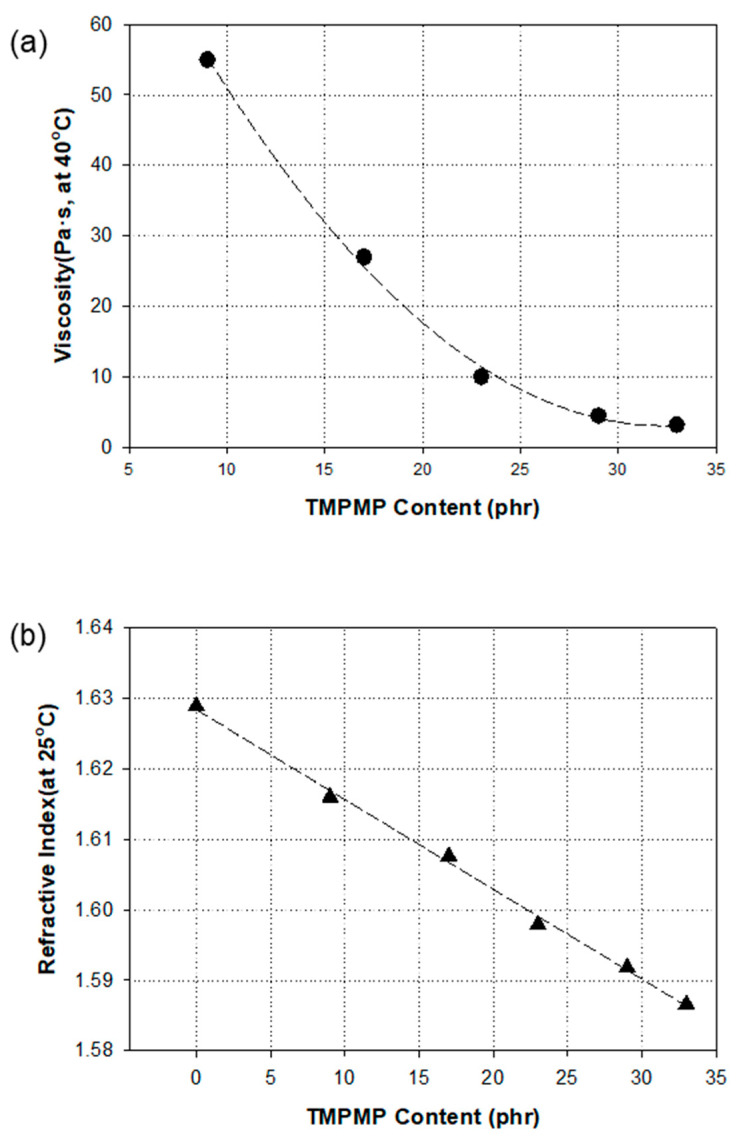
Viscosity (**a**) and refractive index (**b**) as a function of TMPMP contents in BPEF-DMP.

The monomer, BPEF-DMP, demonstrated a high refractive index of 1.629 and a high viscosity at 25 °C, making it difficult to handle. To address this, we used the thiol monomer, TMPMP, as a reactive diluent to adjust the viscosity of BPEF-DMP. Figure 5 illustrates the changes in viscosity and refractive index when the TMPMP was mixed with BPEF-DMP. As the TMPMP content increased, the refractive index decreased linearly, while the viscosity dropped significantly at lower TMPMP levels. However, at higher TMPMP concentrations, the reduction in viscosity became less pronounced. Even with a 23 phr dilution of the TMPMP in BPEF-DMP, the refractive index of the mixture reached 1.5981, and its viscosity at 40 °C was 9.7 Pa·s. This study used a 23 phr diluted mixture of BPEF-DMP and TMPMP as the basic thiol compound for further formulation, making it easier to handle while achieving an optimal composition of BPEF-DMP. This compound can serve as a reactive thiol monomer in prism films to attain higher refractive indices. Table 2 lists five different formulations prepared by varying the TMPMP and BPEF-DMP contents in the mixture.

#### 3.2.2. Mechanical Properties of the Film

**Figure 6:**
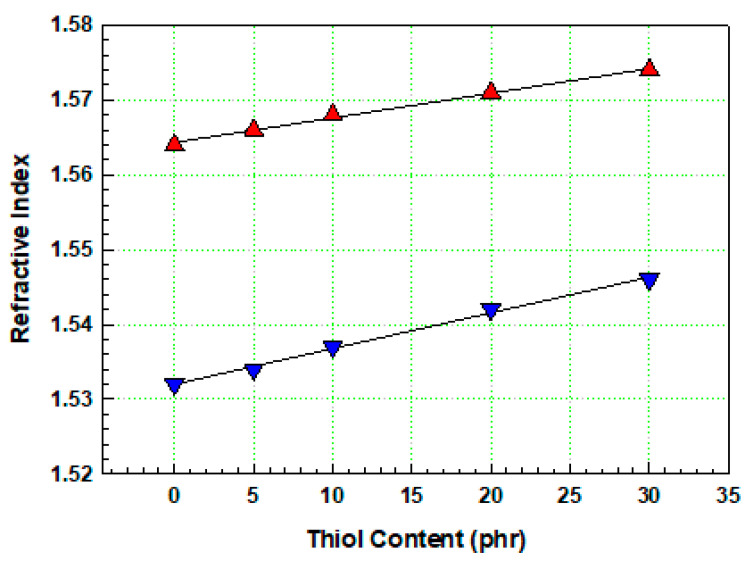
Refractive indices of UV-curable resins before cure (▼) and after cure (▲).

In the formulation without thiol components, the curing reaction proceeded solely through a radical polymerization mechanism initiated by a photoinitiator (TPO), and the refractive index was measured as 1.564. When the thiol components were introduced into the base formulation, crosslinking occurred simultaneously through free radical polymerization and thiol–acrylate reactions. As shown in Figure 6, as the thiol content in the resin increased, the refractive index of mixture E (containing 30 phr thiol) increased to 1.574. For the cured film without thiol components, more than 65% of the coating on the bare PET was delaminated, and the adhesion was rated as 0 B. As the thiol content of the coating increased, adhesion improved significantly. For films with 30 phr thiol, only a small part of the coating was delaminated at the crosshatch points, affecting less than 5% of the area, and the adhesion was rated 4 B. PET, a crystalline polymer, is typically a low-adhesion polymer. Owing to its low surface roughness and challenges in material erosion, adhesion to the coating is typically low, and primer treatment is generally used to improve adhesion to the coating. Primers are typically water-based polyurethane resins, which enhance surface roughness and polarity, increasing adhesion when applied in thicknesses of less than 1 µm. By contrast, primer-treated PET showed better adhesion to the cured layer. Although the increase in adhesion with increasing thiol content was not as pronounced, adhesion improved with increasing thiol content in untreated bare PET, suggesting that the thiol–acrylate reaction during UV curing is an effective method to enhance the adhesion of the coating layer to the untreated bare PET foil. These results highlight the potential for producing cost-effective prism films without primer treatment, offering practical value for industrial applications. UV curing systems based on the thiol–acrylate click reaction are well known for their lower volume shrinkage and shrinkage stress compared to free radical curing reactions due to a delayed gel point transition caused by the step-growth mechanism [28,29,30]. The volume shrinkage of the cured film can be determined using Archimedes’ principle, which utilizes buoyancy in a liquid to measure the density, as shown in the following equation [31]:

(2) $$Shrinkage\left(vol\%\right)=\frac{D_{a}-D_{b}}{D_{a}}\times 10,$$

where D_a_ denotes the density after curing, and D_b_ denotes the density before curing.

As expected, the volume shrinkage of the cured film decreased as the thiol content increased in Table 3. The volume shrinkage decreased from 6.55% to 2.40% as the thiol content increased from 5 phr to 30 phr. The higher the thiol content in the formulation, the more delayed the gelation process during UV curing because of the thiol–acrylate reaction. Therefore, most of the shrinkage in the UV curing system occurred before the gel point. This behavior dramatically reduces the shrinkage stress of the final cured film. The lower shrinkage behavior and higher crosslinking uniformity resulted in improved surface adhesion owing to the reduced stress buildup during film formation [30,32].

#### 3.2.3. Thermal Properties of the Film

**Figure 7:**
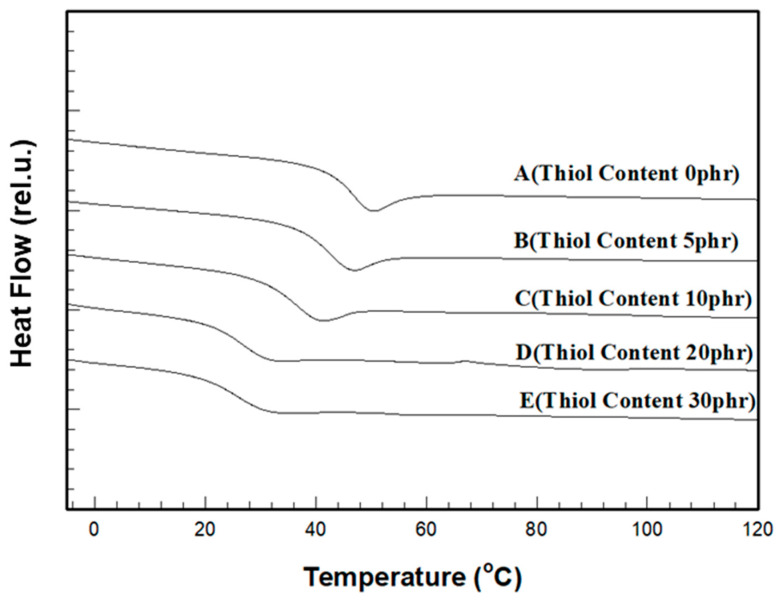
DSC thermograms for various thiol contents in the cured film.

The thermal properties of the cured films after UV curing of the formulations were evaluated using DSC and TGA. The temperature records of the DSC samples showed that as the thiol content increased, the glass transition temperature (Tg) decreased (Figure 7). For the film without thiol, the Tg was 44.02 °C, but for the film containing 30 phr thiol, the Tg was reduced to 25.04 °C, a decrease of approximately 19 °C (Table 4), indicating that, as the thiol content increased, the crosslinking points formed by covalent bonds decreased, and the physical entanglements of the crosslinked chains generated by the two curing mechanisms facilitated the motion of the crosslinked chains within the film, resulting in a rubber-like behavior.

**Figure 8:**
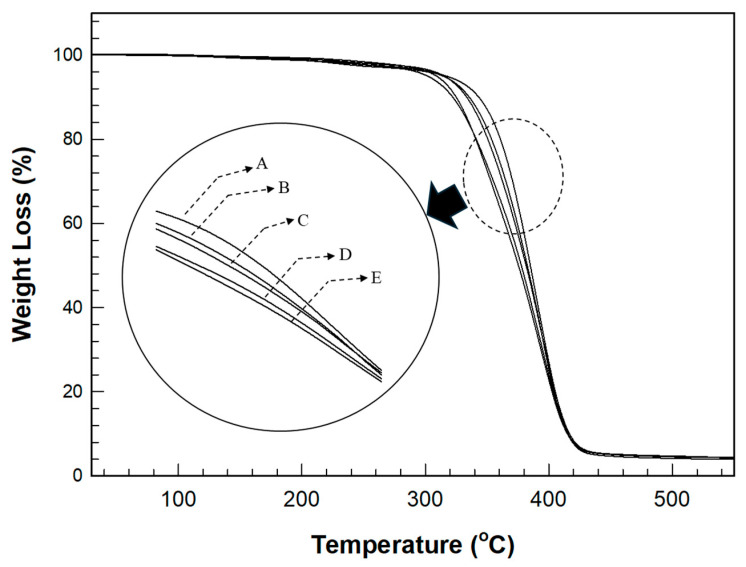
TGA thermograms for various thiol contents in the cured film.

The TGA behavior in Figure 8 shows a trend similar to that of the change in Tg observed in the DSC analysis. As the thiol content increased, the residual weight decreased with increasing temperature; however, the change was less pronounced than the variation in Tg. The temperature at 10% weight loss was 385.01 °C for the film without thiol and 363.51 °C for the film with 30 phr thiol, showing a decrease of 21.5 °C. However, the temperature range of this change was smaller than that observed for Tg. Particularly, the temperature at 50% weight loss was 427.21 °C for the film without thiol and 419.30 °C for the film with 30 phr thiol, a decrease of 8 °C. The range of the temperature change at 50% weight loss was even smaller than that at 10% weight loss.

The cured material formed through the thiol–acrylate reaction exhibited rubber-like properties as the thiol content increased, leading to a decrease in Tg. However, in terms of the high-temperature decomposition of the cured film, even though the film with a higher thiol content showed more rubber-like behavior, it did not show a significant difference in thermal stability compared to the cured film with a lower thiol content, indicating that the cured films with higher thiol contents maintained excellent heat resistance despite their rubber-like characteristics.

### 3.3. Fabrication and Characterization of the Prism Film

#### 3.3.1. Geometrical Properties of the Prism Film

**Figure 9:**
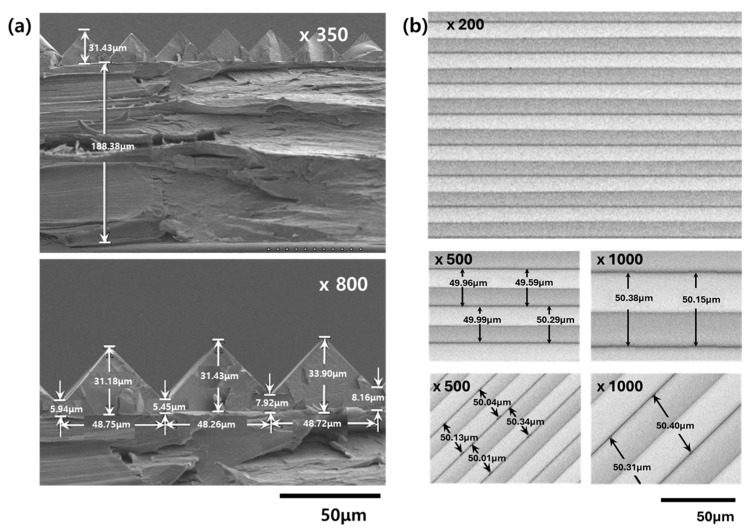
(**a**) Cross-sectional FE-SEM images of the prism film. (**b**) Top view FE-SEM images of the prism film.

The prism film was manufactured using the imprint method through UV curing of the liquid resin. A replica mold, UV-curable resin, and PET foil were placed sequentially and, after lamination, the laminate was irradiated through the PET side to initiate curing. The PET foil was then peeled off to produce a prismatic film. The cross section of the replicated prism film was examined using FE-SEM, and the results are shown in Figure 9. For the 188 μm PET foil, the base layer was 5–8 μm, the height of the prism peaks was 24–26 μm, and the length of the base of the peaks was 48–49 μm, indicating that the replication was relatively uniform and well executed. The top view of the replicated film is shown in Figure 9. The distance between the peaks or valleys was also approximately 50 μm, confirming that the replication was successful and uniform.

#### 3.3.2. Physical Properties

There is a risk that the prism shape may be damaged by vibrations during the transport process or while moving the liquid crystal module during mounting of the prism film in the backlight assembly. Damage to the shape can affect the image quality and decrease the product value of the panel. Research is being conducted to improve the elastic recovery of UV-curable resins to prevent damage to the prism shape, so that, even if physical forces are applied to the prism shape, the shape is not destroyed, and any deformation is quickly restored to its original shape [31,32].

The elastic recovery of the prism-shaped cured layer was measured using a microindenter. A load of 24.5 mN/sec was applied for 10 s to induce displacement, which was denoted as *L*_1_. Subsequently, the same load was removed at the same rate for another 10 s, and the recovered displacement was denoted as *L*_2_. The elastic recovery (*R*) can be defined using the following equation [33], which expresses how much the prism shape recovers after compression deformation:

(3) $$R=(\frac{L_{2}}{L_{1}})\times 100.$$

Typically, UV-cured materials have several crosslink points, making it challenging for them to exhibit viscoelastic behavior. To overcome this limitation, research has been conducted to increase the number of soft segments in the resins used for prism film production, such as large-molecular-weight urethane acrylate resins or acrylate monomers with added ethylene oxide, to reduce the number of crosslink points and enhance the viscoelastic properties [33,34]. However, increasing the number of soft segments in the composition lowers the refractive index of the resin used for film production, reducing the brightness of the prism film.

One of the critical characteristics related to the chemical structure of the cured film and the uniformity of crosslinking in the thiol–acrylate-based network is its physical and mechanical performance. Unlike the free radical polymerization of acrylates, which gels at low conversion, the thiol–acrylate reaction proceeds through a free radical step-growth process, minimizing stress accumulation within the cured material. A reduction in stress increases the elastic modulus of the cured material and improves the adhesion to the substrate. The mechanical properties of thiol-based materials can be attributed to the uniformity of the crosslink densities.

During the free radical polymerization of acrylate monomers and the thiol–acrylate reaction, reports suggest that, when thiol groups exceed a certain conversion (generally slightly below the gel point), the elastic modulus increases [35]. This trend is different from that of the free-radical polymerization of acrylates, where the elastic modulus decreases as the polymerization conversion increases. In the case of thiol–acrylate photopolymerization, the cured network is typically formed at a much higher conversion than the gel formation observed in multifunctional acrylate monomer polymerization. The direct advantage of shrinkage occurring in the liquid phase is that the final cross-linked film ultimately has a higher functional group conversion, and the stress embedded in the formed cured film is lower than that in networks based on traditional acrylate monomer polymerization.

In the TFT LCD structure, the prism film was in contact with the lower polarizing film. The lower polarizing film typically has a surface treatment film with a haze ranging from 1% to 30%, and this surface treatment is designed to introduce roughness to increase the haze. Higher haze indicates increased surface roughness, making the prism shape more susceptible to damage. The peaks of the prism shape are particularly vulnerable to damage, and methods to increase the hardness of the cured material to prevent this can damage the polarizing film in contact with the prism film. Therefore, introducing a cured layer with high elastic recovery can prevent damage to the prism film shape during deformation without damaging the polarizing film.

To simulate the destruction of the prism shape due to vibrations between the prism and polarizing films, a measurement method was devised to quantify this property. The maximum load that did not damage the prism film was used to measure its strength. The damaged areas of the prism film were observed as scratches at the edges of the peaks, indicating that the edges of the peaks, which had smaller contact areas, were more susceptible to damage under pressure.

**Figure 10:**
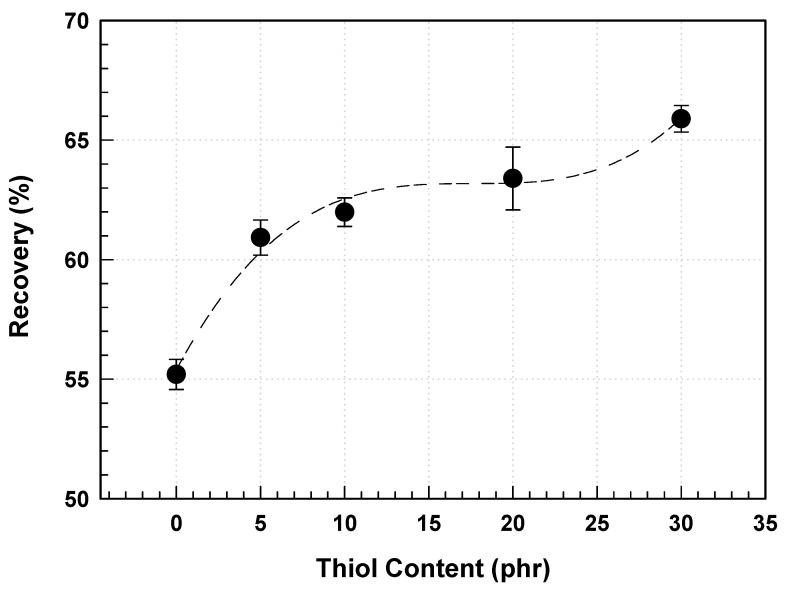
Viscoelastic behavior of prism film as a function of thiol content.

Table 5 shows the strength of the prism film based on the thiol content, with the strength measured as the weight, categorized by the haze of the polarizing film. For a polarizing film with haze, the strength of the prism film with 30 phr thiol ranged from 300 to 1000 g, whereas the films without thiol showed lower strengths of 10 to 100 g. As expected, the strength of the prism film exhibited a trend similar to that of the elastic recovery, increasing with thiol content, indicating that the viscoelastic behavior of the cured material is a favorable factor in reducing the damage to the prism shape (Figure 10). This information is valuable for designing stronger prism films.

#### 3.3.3. Optical Properties

To investigate the impact of thiol content on the overall luminance and color distribution of a BLU, an edge-lit LCD BLU prototype (100 × 100 mm) was fabricated. The LCD BLU comprises a blue LED light source, a diffusion film that homogenizes the light emitted from the light source, and an LGP with a prism sheet sequentially laminated on top of the BLU.

When a prism sheet with a cured layer containing thiol compounds was laminated onto the top of the LCD BLU, the luminance increased as the thiol content increased. When the thiol content of the UV-curable resin was increased to 30 phr, the luminance was 3.96% higher than that of the UV-curable resin without thiol compounds because the increasing thiol content raises the liquid and cured material’s refractive indices. When the refractive index of the cured prism film increases, it becomes more effective at concentrating light in the forward direction, leading to a higher luminance.

Table 6 shows the changes in the color coordinates as a function of the thiol content. Changes in the color coordinates within 3/1000 were not perceptible to the human eye. The x-coordinate, which indicates red, changed by △x = 2/1000 when the thiol content was increased to 30 phr compared to the composition without thiol. This change was considered negligible and within the measurement error range. The y-coordinate, which indicates yellow, changed by △y = 4/1000 when the thiol content was increased to 30 phr compared to the composition without thiol. This increase in the yellow value can be attributed to the higher aromatic content of BPEF-DMP, but the difference was insignificant. Therefore, thiol content does not significantly impact the color coordinates.

## 4. Conclusions

We synthesized a new fluorenyl-containing thiol monomer, BPEF-DMP, with a chemical structure designed to produce high-refractive index prism films. BPEF-DMP was synthesized via low-temperature Fischer esterification, as confirmed by FT-IR, HPLC, and LC-MS analyses. The structure of BPEF-DMP and the impurities formed during the reaction were predicted by mass values (*m*/*z*) using LC-MS, with the purity and control of each impurity optimized using a design of experimental (DOE). By increasing the molar ratio of the reactant (3-MPA) and the catalyst (p-TSA), the purity increased, and the contents of impurities decreased (a detailed explanation is depicted in Supplementary Materials). The synthesized BPEF-DMP monomer, containing terminal thiol groups and an aromatic structure, exhibited a high refractive index. The refractive index of the cured material increased with the thiol content of the UV-curable resin. UV-curing experiments showed that the thiol compounds in the resin allowed volume shrinkage to be controlled through delayed gelation, resulting in a uniformly cured coating. This behavior affected the adhesion to the PET foils, both untreated and primer-treated.

Prism films were produced using UV-curable resins containing thiol compounds, with the thiol content varied as a function of the synthesized BPEF-DMP monomer. In terms of optical properties, when the UV light intensity was low, the photoinitiator TPO did not react sufficiently, causing the film to absorb light in the blue region, turning the film yellow, lowering uniformity, and reducing transmission while increasing haze. Additionally, as measured by DSC, the glass transition temperature (Tg) of the thiol-containing cured film showed a decreasing trend as the thiol content increased, demonstrating rubber-like properties. However, TGA revealed that thermal stability did not significantly decrease, even with higher thiol content. While a decrease in Tg and rubber-like behavior was observed, the thermal decomposition temperature was not significantly affected.

The shape and size of the fabricated prism films were confirmed using FE-SEM, which revealed they were well replicated from the mold. Furthermore, by increasing the thiol content, delayed gelation controlled the volume shrinkage. Microindentation tests confirmed elastic recovery when the thiol conversion reached a certain threshold, resulting in improved mechanical properties. The strength of the prism film was tested using polarized films; as the thiol content increased, the strength of the prism film improved, leading to enhanced handling properties, thus demonstrating that improved elastic recovery of the prism film is a crucial factor in preventing shape damage. When applied to edge-lit LED BLUs, the luminance of the prism films increased with thiol content. Despite this increase in luminance, the color coordinates remained relatively constant and were not significantly affected by the thiol content. In summary, the newly synthesized BPEF-DMP monomer and the UV-curable resin produced from it would be effective in manufacturing prism films with high refractive indices. Moreover, these films exhibit excellent optical and physical properties, making them highly suitable for improving the luminance of BLUs.

## Figures and Tables

**Table 1:** Expected chemical structure of each eluted peak analyzed by HPLC and LC-MS.

	Chemical Structure	Classification
(a)	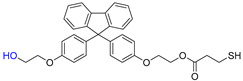	Unreacted product
(b)	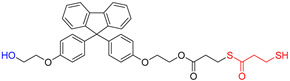	Unreacted product and over-reaction
(c)	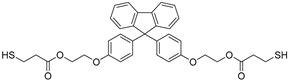	Target product
(d)	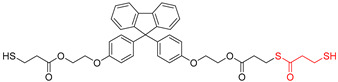	Over-reaction

**Table 2:** Ingredients and formulations of UV-curable resin to fabricate a prism film.

Ingredients	Composition of Mixtures				
	A	B	C	D	E
Thiol * (g)	0	5.0	10.0	20.0	30.0
BPEF-EDA (g)	60.0	60.0	60.0	60.0	60.0
BZA (g)	38.0	38.0	38.0	38.0	38.0
TPO (g)	2.0	2.0	2.0	2.0	2.0

Thiol *: BPEF-DMP 100/TMPMP 23 mixture.

**Table 3:** Volumetric shrinkage of the cured film and density of the UV-curable resin before and after curing.

Entry	Density		Shrinkage (%)
	Before Cure	After Cure	
A	1.1442	1.2369	7.495
B	1.1521	1.2321	6.493
C	1.1604	1.2262	5.366
D	1.1712	1.2191	3.929
E	1.1902	1.2154	2.073

**Table 4:** Thermal characteristics of the thiol-containing UV-cured film.

Entry	DSC Analysis		TGA Analysis	
	Tg_o_ (°C) ^(1)^	Tg_m_ (°C) ^(2)^	T_d10_ (°C) ^(3)^	T_d50_ (°C) ^(4)^
A	41.88	44.02	385.01	427.21
B	36.03	39.22	377.62	425.01
C	29.71	33.79	375.60	424.12
D	20.60	25.83	366.29	421.43
E	19.01	25.04	363.51	419.30

^(1)^ Tg_o_: Glass transition temperature at onset. ^(2)^ Tg_m_: Glass transition temperature at midpoint. ^(3)^ T_d10_: Temperature at 10% wt. loss. ^(4)^ T_d50_: Temperature at 50% wt. loss.

**Table 5:** Strength of the prism film as a function of polarizer film haze and thiol content.

Entry	Polarizer Film Haze (%)			
	5	15	25	30
A	500 g	100 g	50 g	30 g
B	800 g	200 g	200 g	50 g
C	1000 g	500 g	300 g	100 g
D	1400 g	800 g	400 g	200 g
E	1800 g	1000 g	800 g	400 g

**Table 6:** Average color combination and luminance change as a function of content of thiol component in the UV-curable resin.

Entry	Color Coordination		Luminance
	△x	△y	△L (%)
A	Reference	Reference	Reference
B	1/1000	1/1000	100.98
C	2/1000	3/1000	102.44
D	2/1000	3/1000	103.08
E	2/1000	4/1000	103.96

## Data Availability

The original contributions presented in this study are included in the article. Further inquiries can be directed to the corresponding author.

## Supplementary Materials

The following supporting information can be downloaded at: https://www.mdpi.com/article/10.3390/polym17010076/s1, Figure S1. HPLC chromatogram of EXP1 condition (3-MPA 2.0 mol/catalyst 0.015 mol) (a) Unreacted product, (b) Unreacted product & over-reaction, (c) Target product, (d) Over-reaction); Figure S2. HPLC chromatogram of EXP2 condition (3-MPA 2.0 mol/catalyst 0.005 mol) (a) Unreacted product, (b) Unreacted product & over-reaction, (c) Target product, (d) Over-reaction); Figure S3. HPLC chromatogram of EXP3 condition (3-MPA 2.4 mol/catalyst 0.015 mol) (a) Unreacted product, (b) Unreacted product & over-reaction, (c) Target product, (d) Over-reaction); Figure S4. HPLC chromatogram of EXP4 condition (3-MPA 2.4 mol/catalyst 0.005 mol) (a) Unreacted product, (b) Unreacted product & over-reaction, (c) Target product, (d) Over-reaction); Figure S5. HPLC chromatogram of EXP5 condition (3-MPA 2.2 mol/catalyst 0.01 mol) (a) Unreacted product, (b) Unreacted product & over-reaction, (c) Target product, (d) Over-reaction); Figure S6. Main effect analysis for each synthesis DOE factor; Table S1: DOE condition of optimizing for synthesis BPEF-DMP; Table S2: The composition of the Fischer esterification reaction, summary of the chromatographic analysis after the reaction; Table S3. Calculation of each response and their SH value. Reference [36] are cited in the Supplementary Materials.
