# Supplementary material for: Synthesis and Characterization of UV-Curable Resin with High Refractive Index for a Luminance-Enhancing Prism Film
Source: Polymers (Basel). 2024 Dec 30;17(1):76. doi: 10.3390/polym17010076 (PMC11723285; doi:10.3390/polym17010076)
Supplement: Supplementary file 1 [file polymers-17-00076-s001.zip › polymers-3376403-supplementary.pdf]

<Supporting information>

Synthesis and Characterization of UV-Curable Resin with High Refractive Index for a Luminance-Enhancing Prism Film

[1,2]Jin Han Song, [2]Seung-Mo Hong, [2]Seok Kyu Park, [1]Hyeok Ki Kwon, [3]Seok-Ho Hwang, [4]Jong-Min Oh, [4]Sang-Mo Koo, [1]Giwon Lee*, [1]Chulhwan Park*

[1]Department of Chemical Engineering, Kwangwoon University, 20, Kwangwoon-ro, Nowon-gu, Seoul, 01897, Republic of Korea

[2]R&D Center, SHIN-A T&C, 184 Gasan Digital 2-ro, Geumcheon-gu, Seoul, 0851, Republic of Korea

[3]School of Polymer System Engineering, Dankook University, 152 Jukjun-ro, Suji-gu, Yongin, Gyeonggi-do, 16890, Republic of Korea

[4]Department of Electronic Materials Engineering, Kwangwoon University, Seoul, 01897, Republic of Korea

- ***Optimization of BPEF-DMP synthesis***

To optimize the final product through experimentation, it is necessary to consider many variables in order to achieve good results. However, it is not easy to determine which specific properties of the final product are closely related to which variables. Therefore, to conduct efficient experiments and analyze the results, it is crucial to thoroughly plan the experiment in advance so that reliable analysis of the data can be performed. Research on experimental design, which allows for scientific analysis of results through joint research by statisticians and scientists, has been widely conducted. This approach is known as Design of Experiment (DOE), and although it originated in agricultural and life sciences, it has recently been widely applied in engineering fields as well [1].

In this study, among the various methods of DOE, Factorial Design was used to optimize the synthesis of BPEF-DMP. Factorial design is a method in which experiments are performed for every combination of levels of each factor, and the order of the experiments is randomized. Depending on the number of factors, factorial design can be classified as one-way, two-way, or multi-way. If there are n factors, and each factor has 2 levels, then 2^n experiments are needed, while if each factor has 3 levels, 3^n experiments are required. In this study, the factors involved in the reaction between BPEF and 3-MPA were the reaction temperature, reaction time, the mole ratio of the reactants, and the amount of catalyst, thus there were four factors to consider. However, the reaction temperature is a fixed factor since it is maintained at the boiling point of toluene during the azeotropic distillation, and the reaction time is not a parameter that needs optimization since the reaction can be terminated when the theoretical amount of water (byproduct) is released. Therefore, this study focused on optimizing the mole ratio of the reactants and the amount of catalyst as factors, to identify the optimal conditions for the synthesis of BPEF-DMP through DOE.

In this DOE, the purity of the product was the main variable of interest, and it was important to determine which of the selected factors (mole ratio of the reactants and amount of catalyst) had the greatest impact on the purity. Each factor had 2 levels, and a full factorial design was applied, which involved 4 experiments to cover all the possible combinations of the factors. The full factorial design

allows for efficient collection of useful information while minimizing the number of experiments. Additionally, center points were added in this DOE to reduce experimental errors and further confirm the effects of the factors at different levels. **Table S1** summarizes the factors and their levels used in this study. The mole numbers of 3-MPA and the catalyst were based on 1 mol of BPEF.

There are various synthesis parameters in the Fischer esterification reaction, but the mole ratio of the reactants and the concentration of the catalyst are the factors that can be directly controlled. Therefore, experiments were conducted according to the DOE with two factors: the mole ratio of the reactants and the concentration of the catalyst, to optimize the synthesis conditions for BPEF-DMP (see **Table S1**). However, determining the purity of the reaction products is challenging, so LC-MS was used to predict the chemical structures of the products and impurities based on the mass values of the peaks detected. Additionally, HPLC was employed to measure the area ratios of each peak and determine the response values (Y values). **Figures S1-S5** show the HPLC charts for each experimental condition, and **Table S2** summarizes the area ratios of the four peaks under each experimental condition. The area of each peak in the HPLC chromatogram was defined as follows:

Y1 = (a) + (b) peak area for byproducts containing unreacted hydroxyl groups,

Y2 = (b) + (d) peak area for overreaction by-products formed due to thiol-ester reactions,

Y3 = (c) peak area for the purity of the synthesized product.

The calculated Y1, Y2, and Y3 values are summarized in **Table S3**, along with the SH values measured by terminal group analysis. However, the SH values did not significantly affect the impurity content or purity. Y1 and Y2 values are related to impurity content, where smaller values are preferable (smaller the better), while Y3, representing purity, should be as large as possible (larger the better).

Figure S6 presents the main effect analysis for the response variables Y1, Y2, and Y3 with respect to the amounts of 3-MPA and the catalyst (p-TSA). For the response related to unreacted water (Y1), both the amount of 3-MPA and the amount of p-TSA showed a significant negative slope, indicating that increasing the amounts of both factors reduced the Y1 value, which is a smaller the better

characteristic. For the overreaction response (Y2), a positive slope was observed for the amount of 3-MPA, meaning that decreasing the amount of 3-MPA helped reduce Y2, which is also a smaller the better characteristic. The amount of p-TSA had little effect on Y2, so it can be concluded that the main factor influencing Y2 is the amount of 3-MPA. For the response related to purity (Y3), both the amount of 3-MPA and the amount of p-TSA showed positive slopes, meaning that increasing their amounts helped improve the purity, which is a larger the better characteristic. It was found that the effect of 3-MPA on Y3 was more pronounced than that of p-TSA, but since p-TSA also had a relatively significant slope, it cannot be ignored.

From the analysis of Y1, Y2, and Y3, it was determined that the level of overreaction impurities (Y2) was minimal and negligible. To optimize the responses for Y1 and Y2, increasing both the amount of 3-MPA and the catalyst (p-TSA) was found to reduce impurities and improve purity. However, increasing the amounts of raw materials indefinitely would be economically unfeasible, so future research should consider both the input quantities and the economic feasibility in a more comprehensive way.

Table S1. DOE condition of optimizing for synthesis BPEF-DMP

No.	Factor	
	**3-MPA Amount**	**Catalyst Amount**
**EXP1**	**Low Level (-)**<br>2.0 mol	**High Level (+)**<br>0.015 mol
**EXP2**	**Low Level (-)**<br>2.0 mol	**Low Level (-)**<br>0.005 mol
**EXP3**	**High Level (+)**<br>2.4 mol	**Low Level (-)**<br>0.015 mol
**EXP4**	**High Level (+)**<br>2.4 mol	**High Level (+)**<br>0.005 mol
**EXP5**	**Middle Level (0)**<br>2.2 mol	**Middle Level (0)**<br>0.01 mol

Table S2. The composition of the Fischer esterification reaction, summary of the chromatographic analysis after the reaction

Entry	Reactant composition (mol)			Peak area integral (%)			
	**BPEF**	**3-MPA**	***p*-TSA**	(a)	(b)	(c)	(d)
EXP1	1.0	2.0	0.015	6.588	0.560	91.791	1.061
EXP2	1.0	2.0	0.005	17.125	1.250	81.159	0.465
EXP3	1.0	2.4	0.015	1.588	2.217	94.616	1.579
EXP4	1.0	2.4	0.005	4.393	2.431	92.314	0.862
EXP5	1.0	2.2	0.010	5.572	1.754	91.680	0.994

Table S3. Calculation of each response and their SH value

Entry	Response			
	Y1 (%)	Y2 (%)	Y3 (%)	SH value (g/eq.)
EXP1	7.148	1.621	91.791	339.96
EXP2	18.375	1.715	81.159	340.12
EXP3	3.805	3.796	94.616	340.62
EXP4	6.824	3.293	92.314	339.87
EXP5	7.326	2.748	91.680	340.87

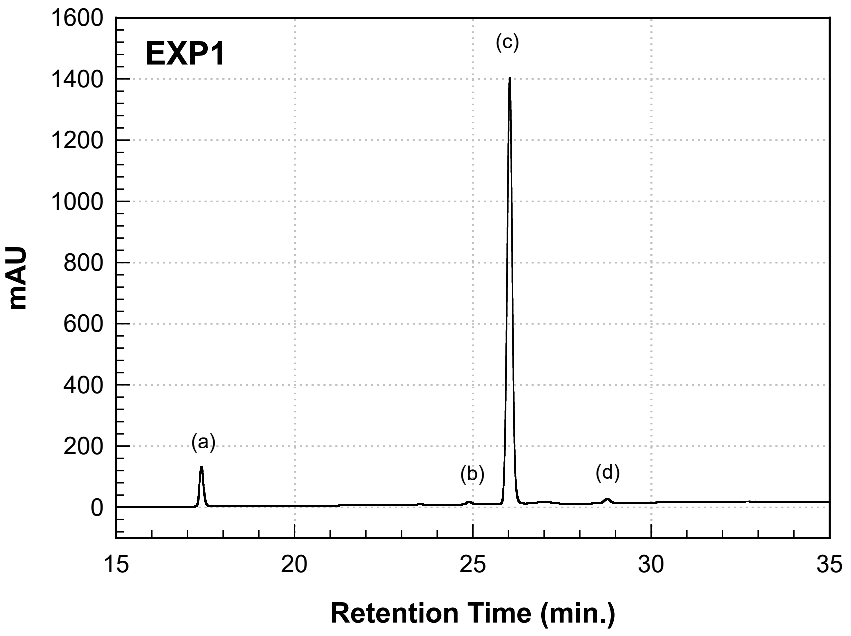

Figure S1. HPLC chromatogram of EXP1 condition (3-MPA 2.0 mol / catalyst 0.015 mol)

(a) Unreacted product, (b) Unreacted product & over-reaction, (c) Target product, (d) Over-reaction)

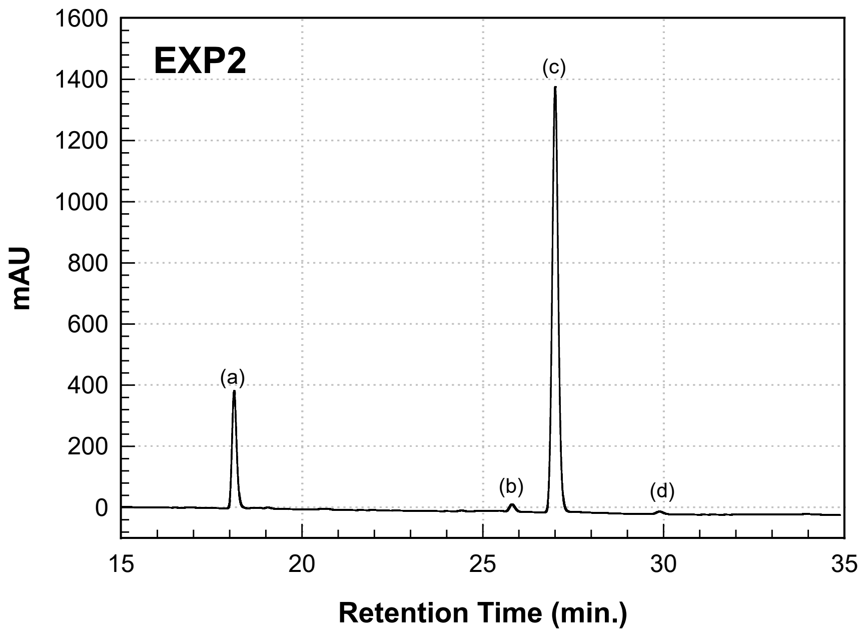

Figure S2. HPLC chromatogram of EXP2 condition (3-MPA 2.0 mol / catalyst 0.005 mol)

(a) Unreacted product, (b) Unreacted product & over-reaction, (c) Target product, (d) Over-reaction)

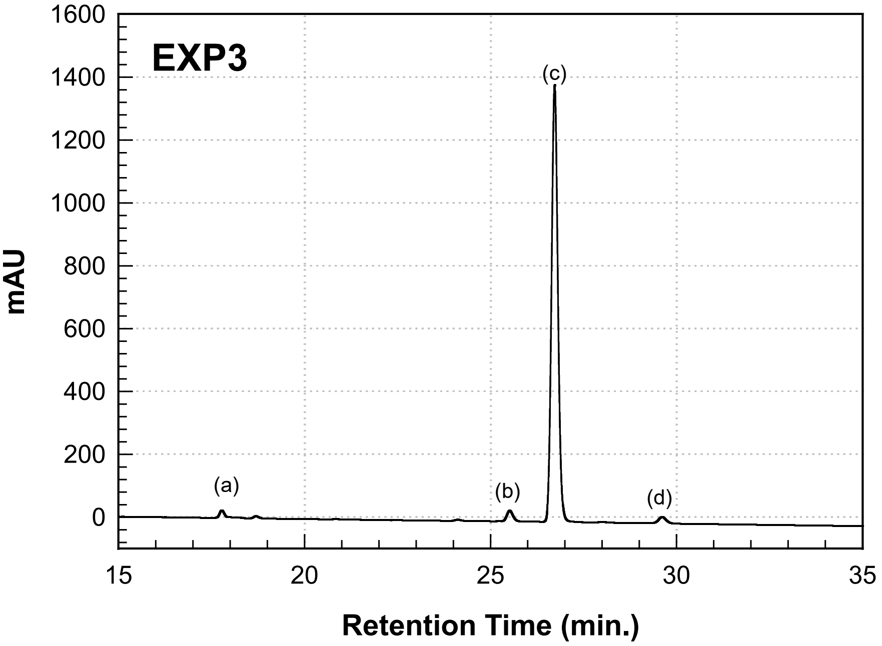

Figure S3. HPLC chromatogram of EXP3 condition (3-MPA 2.4 mol / catalyst 0.015 mol)

(a) Unreacted product, (b) Unreacted product & over-reaction, (c) Target product, (d) Over-reaction)

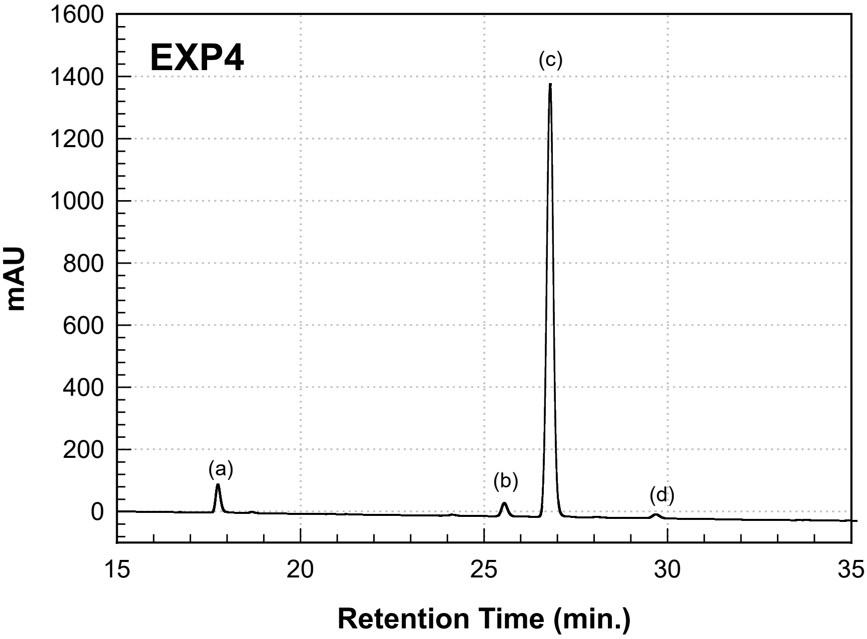

Figure S4. HPLC chromatogram of EXP4 condition (3-MPA 2.4 mol / catalyst 0.005 mol)

(a) Unreacted product, (b) Unreacted product & over-reaction, (c) Target product, (d) Over-reaction)

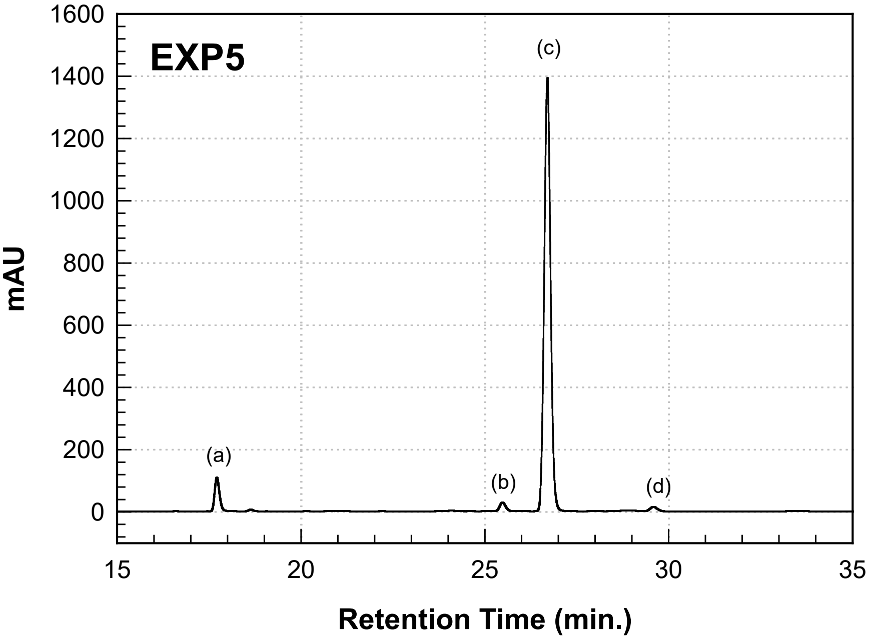

Figure S5. HPLC chromatogram of EXP5 condition (3-MPA 2.2 mol / catalyst 0.01 mol)

(a) Unreacted product, (b) Unreacted product & over-reaction, (c) Target product, (d) Over-reaction)

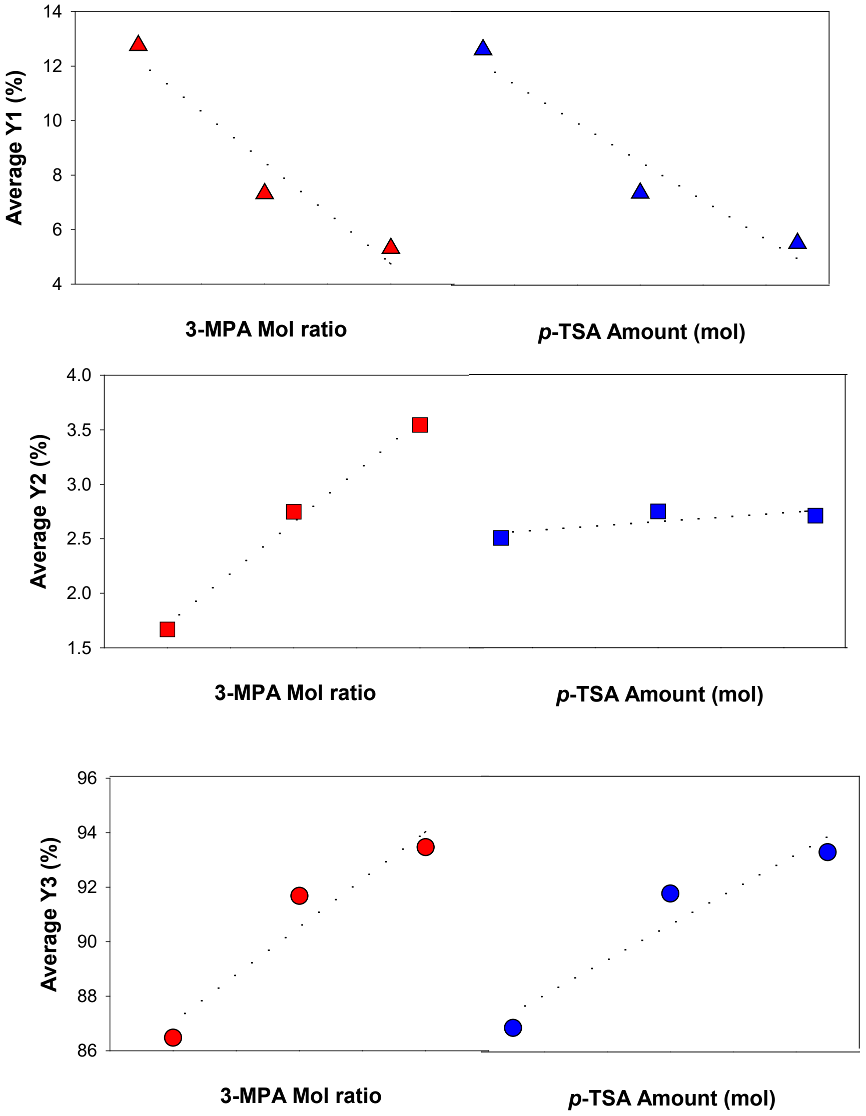

Figure S6. Main effect analysis for each synthesis DOE factor

References

[35] R. A. Fisher, “Design of Experiments” *Br. Med. J.*, *1*(3923): 554., 1936
